# Alterations in synapses and mitochondria induced by acute or chronic intermittent hypoxia in the pre-Bötzinger complex of rats: an ultrastructural triple-labeling study with immunocytochemistry and histochemistry

**DOI:** 10.3389/fncel.2023.1132241

**Published:** 2023-06-16

**Authors:** Junjun Kang, Naining Lu, Shoujing Yang, Baolin Guo, Yuanyuan Zhu, Shengxi Wu, Xiaofeng Huang, Margaret T. T. Wong-Riley, Ying-Ying Liu

**Affiliations:** ^1^Department of Neurobiology, The Fourth Military Medical University, Xi’an, China; ^2^Department of Pathology, The Fourth Military Medical University, Xi’an, China; ^3^Department of Pathology, Xi’an Gaoxin Hospital, Xi’an, China; ^4^Department of Cell Biology, Neurobiology and Anatomy, Medical College of Wisconsin, Milwaukee, WI, United States

**Keywords:** neuroplasticity, synapse, ultrastructure, mitochondria, desmosome-like contact, intermittent hypoxia, pre-Bötzinger complex

## Abstract

**Introduction:**

The pre-Bötzinger complex (pre-BötC), a kernel of inspiratory rhythmogenesis, is a heterogeneous network with excitatory glutamatergic and inhibitory GABAergic and glycinergic neurons. Inspiratory rhythm generation relies on synchronous activation of glutamatergic neuron, whilst inhibitory neurons play a critical role in shaping the breathing pattern, endowing the rhythm with flexibility in adapting to environmental, metabolic, and behavioral needs. Here we report ultrastructural alterations in excitatory, asymmetric synapses (AS) and inhibitory, symmetric synapses (SS), especially perforated synapses with discontinuous postsynaptic densities (PSDs) in the pre-BötC in rats exposed to daily acute intermittent hypoxia (dAIH) or chronic (C) IH.

**Methods:**

We utilized for the first time a combination of somatostatin (SST) and neurokinin 1 receptor (NK1R) double immunocytochemistry with cytochrome oxidase histochemistry, to reveal synaptic characteristics and mitochondrial dynamic in the pre-BötC.

**Results:**

We found perforated synapses with synaptic vesicles accumulated in distinct pools in apposition to each discrete PSD segments. dAIH induced significant increases in the PSD size of macular AS, and the proportion of perforated synapses. AS were predominant in the dAIH group, whereas SS were in a high proportion in the CIH group. dAIH significantly increased SST and NK1R expressions, whereas CIH led to a decrease. Desmosome-like contacts (DLC) were characterized for the first time in the pre-BötC. They were distributed alongside of synapses, especially SS. Mitochondria appeared in more proximity to DLC than synapses, suggestive of a higher energy demand of the DLC. Findings of single spines with dual AS and SS innervation provide morphological evidence of excitation-inhibition interplay within a single spine in the pre-BötC. In particular, we characterized spine-shaft microdomains of concentrated synapses coupled with mitochondrial positioning that could serve as a structural basis for synchrony of spine-shaft communication. Mitochondria were found within spines and ultrastructural features of mitochondrial fusion and fission were depicted for the first time in the pre-BötC.

**Conclusion:**

We provide ultrastructural evidence of excitation-inhibition synapses in shafts and spines, and DLC in association with synapses that coincide with mitochondrial dynamic in their contribution to respiratory plasticity in the pre-BötC.

## Introduction

Neural networks are executed through structural and functional plasticity of synapses to adapt to continuous environmental changes (Citri and Malenka, 2008). Alterations in the synaptic strength with either increased or decreased synaptic plasticity are thought to represent the cellular mechanisms of learning and memory, characterized as long-term potentiation (LTP, Abbott and Nelson, 2000; Sutton et al., 2006). Synaptic plasticity is tightly correlated with structural changes such as the larger postsynaptic density (PSD) area and spine volume, and increases in perforated PSDs in hippocampal and cortical synapses (Toni et al., 2001; Stewart et al., 2005; Holler et al., 2021). While synaptic plasticity has largely focused on excitatory glutamatergic activity, inhibitory GABAergic synaptic plasticity is also receiving attention, thereby excitatory and inhibitory synapses are assumed to co-orchestrate in contribution to neuroplasticity (Chiu et al., 2013, 2019; Ravasenga et al., 2022). Long-term facilitation (LTF), characterized as a progressive and sustained increase in respiratory motor output lasting for hours, is induced by a moderate, acute intermittent hypoxia (AIH), which represents a form of the respiratory system plasticity (Mitchell et al., 2001; Baker-Herman and Mitchell, 2002). Preconditioning animals with a daily AIH (dAIH) can promote respiratory and motor functional recovery after spinal cord injuries (Gonzalez-Rothi et al., 2015; Vose et al., 2022). Conversely, a severe chronic intermittent hypoxia (CIH), which mimics the recurrent episodes of obstructive sleep apnea (OSA) in patients, causes detrimental consequences (Del Rio et al., 2016). However, ultrastructural evaluation of synaptic characteristics, and excitatory and inhibitory synaptic relationship relevant to respiratory plasticity remains little investigated.

The pre-Bötzinger complex (pre-BötC), a presumed kernel essential for inspiratory rhythmogenesis in the ventrolateral medulla, is involved in respiratory plasticity (Barnett et al., 2017; Garcia et al., 2019). CIH imparts inspiratory rhythmogenesis in a state-dependent manner in the isolated pre-BötC, leading to a loss of transmission fidelity in the local respiratory circuit (Garcia et al., 2016, 2017). Regardless of the electrophysiological information, knowledge of synaptic ultrastructural alterations, including the PSD area and complexity, excitatory and inhibitory balance, and dendritic spine features is essential for the understanding of synaptic strength, efficacy, and plasticity. However, synapse-specific ultrastructural modifications in response to respiratory plasticity have not been precisely characterized in the pre-BötC.

Pre-BötC neurons are heterogeneous and composed of excitatory glutamatergic and inhibitory GABAergic and glycinergic neurons (Kuwana et al., 2006; Wallén-Mackenzie et al., 2006; Winter et al., 2009). Neurokinin 1 receptors (NK1R) and somatostatin (SST) are highly expressed in some of glutamatergic neurons, thereby being used as markers of the pre-BötC in animals (Guyenet and Wang, 2001; Stornetta et al., 2003; Gray et al., 2010) and humans (Schwarzacher et al., 2011). We have demonstrated that SST is colocalized with NK1R in small fusiform and some medium-sized NK1R-positive (NK1R^+^) neurons, and SST^+^ terminals form asymmetric synapses (AS) and symmetric synapses (SS) with NK1R^+^ neurons in the pre-BötC in normoxic (NOR) rats (Wei et al., 2012). Here, with a combination of SST and NK1R double immunocytochemistry and cytochrome oxidase (CO) histochemistry, we sought to determine ultrastructural changes in synapses and mitochondrial dynamics in the pre-BötC in rats preconditioned with dAIH or CIH. We analyzed excitatory and inhibitory synaptic parameters in dendrites, especially perforated synapses that have been assumed to be associated with synaptic efficiency and plasticity (Toni et al., 2001; Stewart et al., 2005). In particular, we characterized a synapse-specific compartment, the spine-shaft microdomain and its association with mitochondria. Desmosome-like contacts (DLC) were featured for the first time in the pre-BötC. Moreover, we documented ultrastructural characteristics of mitochondrial fusion and fission. We provided ultrastructural basis for possible synchronization between synapses and mitochondria in a compartment-specific manner within dendritic shafts and spines in their contribution to respiratory plasticity in the pre-BötC.

## Materials and methods

### Animals and intermittent hypoxic treatments

Adult Sprague-Dawley rats (230–250 g) were housed in a room with a constant temperature and a 12 h light/dark cycle. Water and food were available *ad libitum*. All experimental procedures were approved by the Northwest China Committee of Experimental Animal Care and conformed to the Guide for the Care and Use of Laboratory Animals published by the National Institutes of Health (NIH). All efforts were made to minimize animal suffering and to reduce the number of animals used.

Animals were randomly divided into NOR, dAIH and CIH groups (*n* = 18 for each group). The hypoxic animals were kept in an acrylic chamber for normobaric hypoxia. Hypoxic conditions were established with a mixture of room air and pure N_2_, and monitored by an oxygen analyzer (Attendor 120, Innovation Instrument CO., LTD, Ningbo, China). The dAIH protocol consisted of 5-min episodes of hypoxia (10% O_2_) interspersed with 5-min room air intervals. Changes in O_2_ levels in the chamber were reached within 20 s during hypoxic episodes. Animals experienced dAIH protocol for 2 h at 9:00–11:00 am each day for 2 weeks. For the CIH treatment, levels of oxygen in the chamber were cycling between 21 to 5 ± 0.5% per minute (i.e., 60 hypoxic episodes per hour) for 8 h per day for 10 consecutive days. The chamber was kept at a constant temperature (22 ± 1°C per day for 10 consecutive days. The chaeuthanized 1 h after the last episode of intermittent hypoxia.

### Immunofluorescent histochemistry

Eight animals for each group were deeply anaesthetized with 1% sodium pentobarbital intraperitoneally (50 mg/kg body weight) and were euthanized by transcardial perfusion with 150 ml saline, followed by 500 ml ice cold 4% paraformaldehyde in 0.1 M phosphate buffer (PB, pH 7.4). Brains were removed and post-fixed in the same fixative for 2 h at 4°C. They were then cryoprotected in 30% sucrose in 0.1 M PB overnight at 4°C. Alternate serial coronal sections of brainstems containing the pre-BötC region were cut at 12 μm thickness on a cryostat (CM1900, Leica, Heidelberger, Germany) and mounted on gelatin-coated slides for SST and NK1R immunohistochemistry. All subsequent immunohistochemical procedures were done at room temperature. Slides were blocked and then incubated overnight with a cocktail of primary antibodies of goat anti-SST (1:400, sc-7819, Santa Cruz) and rabbit anti-NK1R (1:5,000, S8305, Sigma, St. Louis, MO) diluted in phosphate buffered saline (PBS) containing 2% bovine serum albumin (BSA) and 0.5% Triton X-100. Secondary antibodies were a mixture of Alexa 488-conjugated anti-goat IgG and Texas Red conjugated anti-rabbit IgG (1:800, Molecular Probes, Eugene, Oregon) in PBS containing 0.3% Triton X-100 for 4 h. After rinsing in PBS, slides were then coverslipped with anti-fading medium and examined with a confocal laser-scanning microscope (Fluoview 1000, Olympus) using laser beams of 543 nm and 488 nm with appropriate emission filters for Texas Red (590–610 nm) and Alexa 488 (510–525 nm), respectively. SST and NK1R immunoreactivities were detected with identical conditions under a microscope equipped with a 40 × objective. Representative photomicrographs were captured with a 12-bit color CCD camera (DP-70, Olympus), acquired at a resolution of 1,360 × 1,024 pixels, and stored in JPEG format.

### CO histochemistry

Four rats from each group were deeply anesthetized and perfused transcardially with 150 ml saline, followed by 50 ml ice-cold mixture of 4% paraformaldehyde and 0.05% glutaraldehyde in 0.1 M PB for 1 h. Brainstems were removed and postfixed by immersion in the same fixative for 4 h at 4mersion in the same fixative for μm thicknesses were prepared with a vibratome (VS1000s, Leica). Sections (*n* = 18–20) including the pre-BötC region were collected from each brainstem. The basic protocol for CO histochemistry was as described previously (Liu et al., 2001). Briefly, sections were incubated in 0.1 M PB containing 25 mg 3, 3’-diaminobenzidine (DAB, Sigma), 15 mg cytochrome c, type III (Sigma), and 2 g sucrose per 50 ml solution. They were incubated at 37 for 3 h in the dark. All sections from three groups were reacted together to avoid differences due to slight variations, such as temperature, medium composition, or incubation time. After CO histochemistry, sections were washed three times, 5 min for each in cold 0.1 M PB. As CO-negative control experiment, two NOR rats were perfused and sectioned as above, and performed SST and NK1R immunocytochemistry procedures, avoiding CO histochemistry.

### Pre-embedding immunogold-silver cytochemistry

CO-reacted sections were then processed for SST and NK1R immunocytochemistry. Sections were blocked for 2 h in PBS containing 5% BSA and 0.05% Triton X-100 and were then incubated overnight in the mixed antibodies of goat anti-SST and rabbit anti-NK1R diluted as described above. After rinsing in PBS, they were then incubated in horseradish peroxidase conjugated anti-goat IgG (1:400, Molecular Probes, Eugene, Oregon) and anti-rabbit IgG conjugated to 1.4 nm gold particles (1:100, Nanoprobes, Stony Brook, NY). Sections were rinsed and postfixed in 2% glutaraldehyde in PBS for 45 min. Signals of NK1R immunoreactivity were detected by silver enhancement kit (HQ Silver Kit, Nanoprobes) in the dark. Prior to, and after silver enhancing, sections were rinsed several times with deionized water. After rinsing in PBS, they were visualized by the glucose oxidase-DAB method for SST immunoreactivity. Sections were postfixed in 0.5% osmium tetroxide in 0.1 M PB for 2 h. They were then dehydrated with graded ethanol, replaced with propylene oxide, and finally embedded in Epon 812 between plastic sheets. After polymerization, flat-embedded sections were examined under the light microscope. Three-four sections containing SST and NK1R immunoreactivities in the pre-BötC were selected, trimmed under a stereomicroscope, and then glued onto blank resin stubs. Serial ultrathin sections were cut with a diamond knife (Diatome, Hatfield, PA) and an Ultramicrotome (Leica EM UC6, Wetzlar, Germany), and mounted on formvar-coated mesh grids (6–8 sections/grid). They were then counterstained and examined under the electron microscope (JEM-1230, JEOL LTD, Tokyo, Japan) equipped with CCD camera and its application software (832 SC1000, Gatan, Warrendale, PA). Electron micrographs (*n* = 80) of postsynaptic dendrites that were labeled by NK1R-immunogold particles and had synapses with distinct synaptic clefts in the pre-BötC from one brainstem, a total of 320 of dendritic profiles from each group were collected for quantitative analysis with Image-Pro Plus software (Media Cybernetics, Inc.). The quantitative analysis was carried out by a person unaware of the protocols. Regions of PSDs were circled and the area was determined with “area measurement” function. The length and area of PSDs, and the distance between the nearest edges of DLC and PSDs (Figure 3F, curve) were calculated and presented as mean ± SEM. Statistical significance was determined by one-way ANOVA, followed by Kruskal–Wallis test (a *post hoc* test) to identify individual comparisons. The closest distances of mitochondria to the center of DLC and PSDs (Figure 3F, straight lines) were analyzed by Mann Whitney U test. *p* < 0.05 was considered significant.

### Western blot analysis

Western blot analysis was performed according to the standard protocol. Tissues from the ventrolateral medulla oblongata in 18 rats (*n* = 6 for each group) were dissected and homogenized. SDS-PAGE was conducted and protein transferred to PVDF membrane. After the membrane was blocked with 5% non-fat milk, goat-anti SST (1:1,500), rabbit anti-NK1R (1:15,000), or mouse anti-β-actin antibody (1:5,000, Sigma) were incubated with protein-loaded membrane at 4°C overnight. Horseradish peroxidase-conjugated anti-goat, anti-rabbit or anti-mouse secondary antibodies were incubated for 1 h at room temperature. Bands were visualized with a Pierce ECL kit (Thermo Fisher, Rockford) and images were analyzed by Image J. All values were expressed as mean ± SEM. *p* < 0.05 was considered significant.

## Results

**FIGURE 1:**
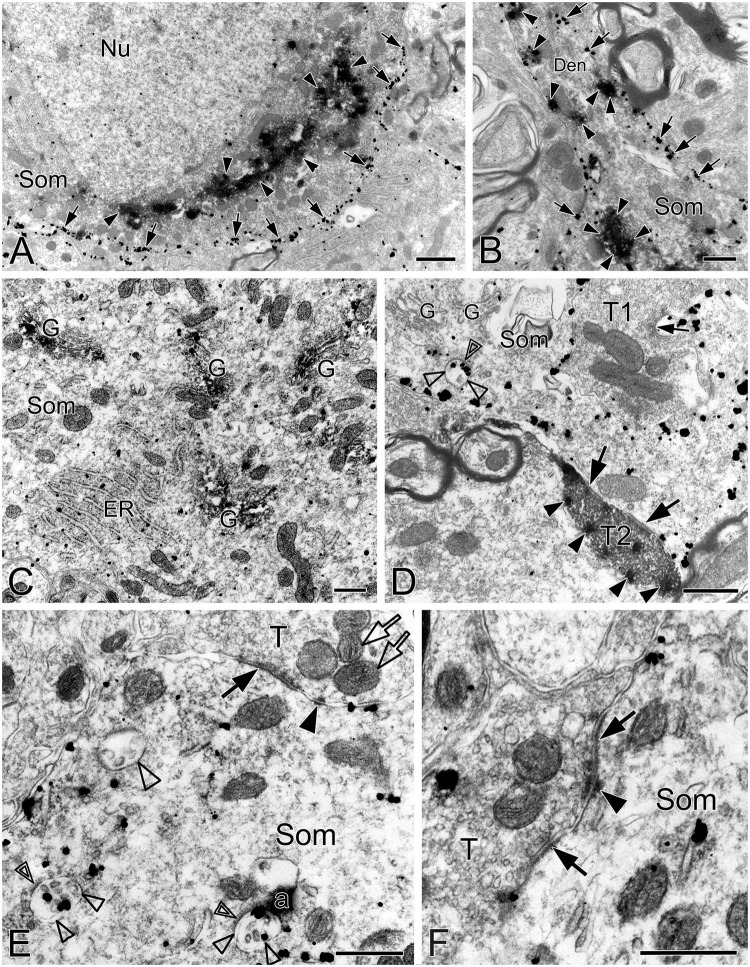
Electron micrographs showing SST and NK1R immunoreactivities in the pre-BötC. NK1R immunogold particles were localized mainly along the inner surface of the plasma membrane [arrows in panels **(A,B)**]. The particles were also distributed in the rough endoplasmic reticulum **(C)** and multivesicular body [open arrowheads in panel **(D)**, open thin arrowheads in panel **(E)**]. Some multivesicular bodies had clathrin coat [double open arrowheads in panels **(D,E)**]. Multivesicular bodies with no NK1R immunoreactivity were also visible [open thick arrowhead in panel **(E)**]. SST immunoperoxidase reaction product was observed in somata [arrowheads in panels **(A,B)**], primary dendrites [arrowheads in panel **(B)**], and large dense-core vesicles in terminals [solid arrowheads in panel **(D)**]. SST was specifically expressed in the Golgi apparatus **(C)**. Perforated symmetric synapses (SS, solid thick arrows in panel **(D,F)**] and horseshoe-shaped SS [solid thin arrow in panel **(D)** were visualized. Desmosome-like contacts (DLC) were seen alongside of SS (solid arrowhead in panel **(E)**] or between two SS [solid arrowhead in panel **(F)**]. Mitochondria seemed in more proximity to DLC [open arrows in panel **(E)**]. Nu: nucleus, Som: soma, ER: endoplasmic reticulum, G: Golgi apparatus, T: terminal, Den: dendrite. a: artificial. Scale bars in panels **(A,B)**: 1 μm, in panels **(C–F)**: 0.5 μm.

**FIGURE 2:**
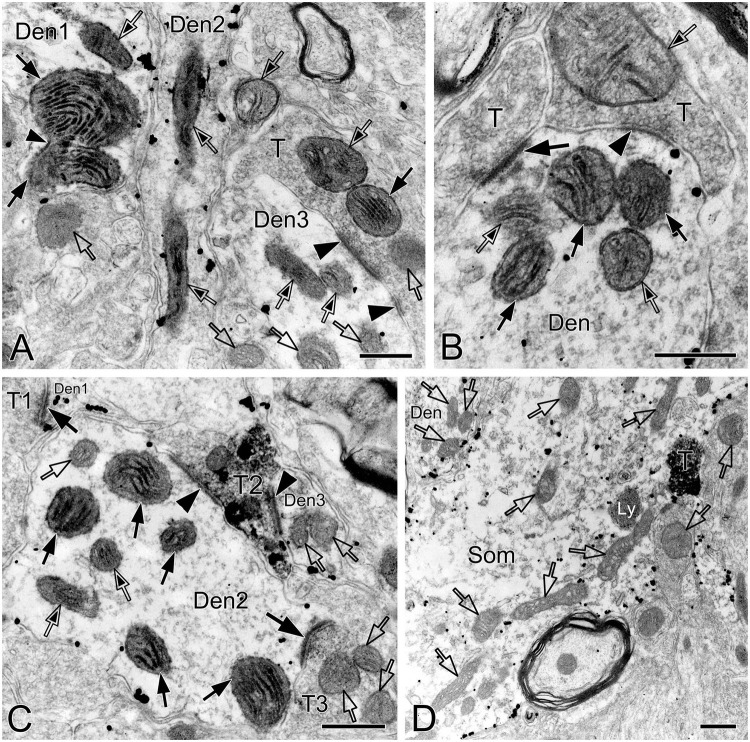
Electron micrographs showing CO-reactive mitochondria in the pre-BötC. Mitochondria with dark (solid thin arrows), moderate (open arrows with solid arrowheads), and light (open arrows) CO reactivity were identified in NK1R^+^ dendrites **(A–C)**. Open arrows in panel **(D)** indicated mitochondria with no CO histochemistry. Thin arrowhead in panel **(A)** pointed to the presumable fused membrane of two mitochondria. Som: soma, Den: dendrite, T: terminal. Ly: lysosome. Scale bars: 0.5 μm.

This was the first time that a triple labeling with a combination of SST and NK1R double immunocytochemistry and CO histochemistry at the ultrastructural level was reported in the pre-BötC. We identified AS and SS established by presynaptic SST^+^ or SST^–^ terminals with postsynaptic NK1R^+^ dendrites in the pre-BötC. NK1R immunogold particles were localized mainly along the inner surface of the plasma membrane in somata and dendrites, as represented in Figure 1. The particles were also found in association with the rough endoplasmic reticulum (Figure 1C) and some multivesicular bodies (thin open arrowheads in Figures 1D, E), indicative of NK1R recycling of synthesis, membrane translocation, endocytosis and degradation, a process that is reminiscent of muscarinic acetylcholine receptor 2 in striatal interneurons (Bernard et al., 1998). SST immunoperoxidase reaction product was distributed in somata (Figures 1A, B), axonal terminals (Figure 1D), and primary dendrites (Figure 1B). In somata, SST^+^ product was specifically expressed in the Golgi apparatus (Figure 1C), consistent with previous descriptions (Johansson, 1978). CO reaction product was expressed on the inner mitochondrial membrane, filling the intracristate space, as showed in Figure 2, consistent with our previous studies (Kang et al., 2019, 2020). Three categories of CO-reactive mitochondria were defined to reflect CO activity, according to previous reports (Wong-Riley, 1989; Wong-Riley et al., 1989): darkly reactive mitochondria with more than 50% of the inner mitochondrial membrane and intracristate space bearing CO reaction product, as showed in Figures 2A–C (thin solid arrows), moderate ones with less than 50% of inner mitochondrial membrane and intracristate space covered with reaction product (open arrows with solid arrowheads in Figures 2A–C), and light ones with little or no detectable reaction product (open arrows in Figures 2A, C). Figure 2D shows mitochondria as CO negative control, with omission of CO histochemistry procedure.

### Synaptic ultrastructural features in dendritic shafts in the pre-BötC

**FIGURE 3:**
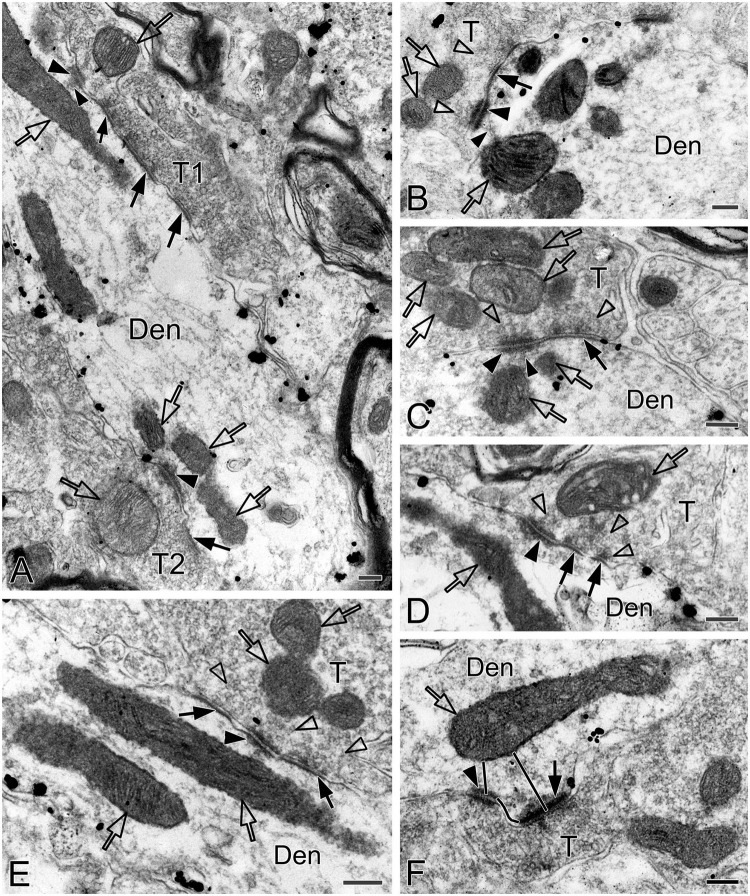
Electron micrographs showing perforated synapses, DLC and mitochondria in the pre-BötC. Perforated synapses had discontinuous postsynaptic densities [PSDs, solid arrows in panels **(A,B,D,E)**]. DLC were found alongside of synapses [solid thick arrowheads in panels **(A–D,F)**] or between two segments of perforated synapses [solid arrowhead in panel **(E)**]. PSDs were seen in continuity to the dense membranes of DLC **(B–D)**. Open arrowheads pointed to synaptic vesicles accumulated in distinct pools opposed each discrete PSD segments and DLC. Open arrows showed mitochondria in proximity to DLC. Filamentous or flocculent elements could be found between the mitochondria and the DLC [solid thin arrowheads in panels **(A–C)**]. Curve in panel **(F)** represented the distance between the DLC and the synapse, and straight lines in panel **(F)** showed the closest distances of mitochondria to the center of DLC and PSDs. Den: dendrite, T: terminal. Scale bars: 0.2 μm.

**FIGURE 4:**
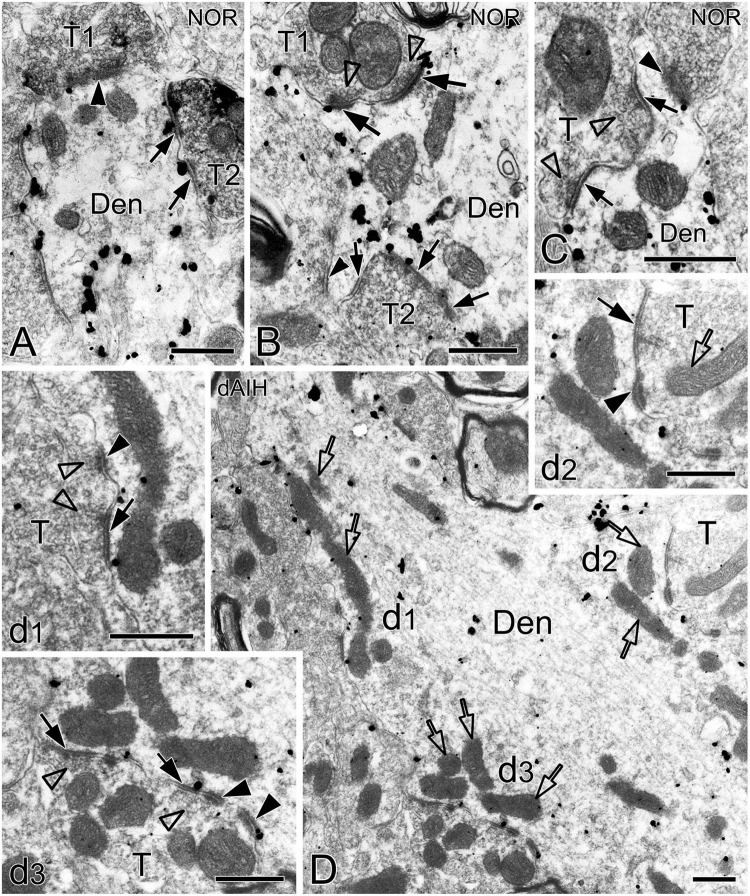
Electron micrographs showing perforated synapses in the pre-BötC. An SST^+^ terminal made perforated SS with a NK1R^+^ dendrite [arrows in panel **(A)**]. Perforated synapses were found to be localized in large dendritic shafts, and also extended into restricted spaces [thin arrows in panels **(B,C)**]. Synaptic vesicles could be seen accumulated in distinct pools opposed each discrete segment of PSDs [open arrowheads in panels (**B,C**,d1,d3)]. Solid arrowheads in panels (d1–d3) pointed to DLC. A mitochondrion was found in more proximity to a DLC than SS [open arrow in panel (d2)]. Areas labeled by panels (d1–d3 in **D**) were magnified in insets. T: terminal, Den: dendrite. Scale bars: 0.5 μm.

Synapses in the pre-BötC were mainly constructed on dendritic shafts (Figures 2A–C, 3, 4, 8), and axo-spinous synapses were rarely detectable (Figure 9). NK1R^+^ dendrites received both AS and SS innervation, as represented in Figure 2. AS are featured with prominent PSDs (thick arrows in Figures 2B, C), corresponding to excitatory synapses, and SS have less pronounced PSDs (arrowheads in Figures 2A–C), referring to inhibitory synapses (Peters et al., 1991). The majority of PSDs had a single plate, termed macular synapses as represented in Figures 2B, C. Perforated synapses, featured by discontinuous PSDs with two or more separate segments were identified in the pre-BötC (thick arrows in Figures 1D, F, solid arrows in 3A with T1, B, D, E, 4). The other characteristic of perforated synapses is their large size, as PSDs with a single perforation are larger than a simple macular one, and the largest have two or more perforations (Peters and Kaiserman-Abramof, 1969; Jones and Harris, 1995). Indeed, multiple perforated synapses with two or more perforations were encountered in the pre-BötC, representing larger and more complex PSDs (solid arrows with T1 in Figure 3A, thin solid arrows with T2 in Figure 4B). Figures 4B, C display perforated AS (thick solid arrows) and SS (thin solid arrows) with curved PSDs that were localized in large dendritic shafts, and also extended into restricted spaces, facing other synapses (solid arrowheads). Synaptic vesicles could be found accumulated in distinct pools opposed each discrete segments of PSDs (open arrowheads).

**FIGURE 5:**
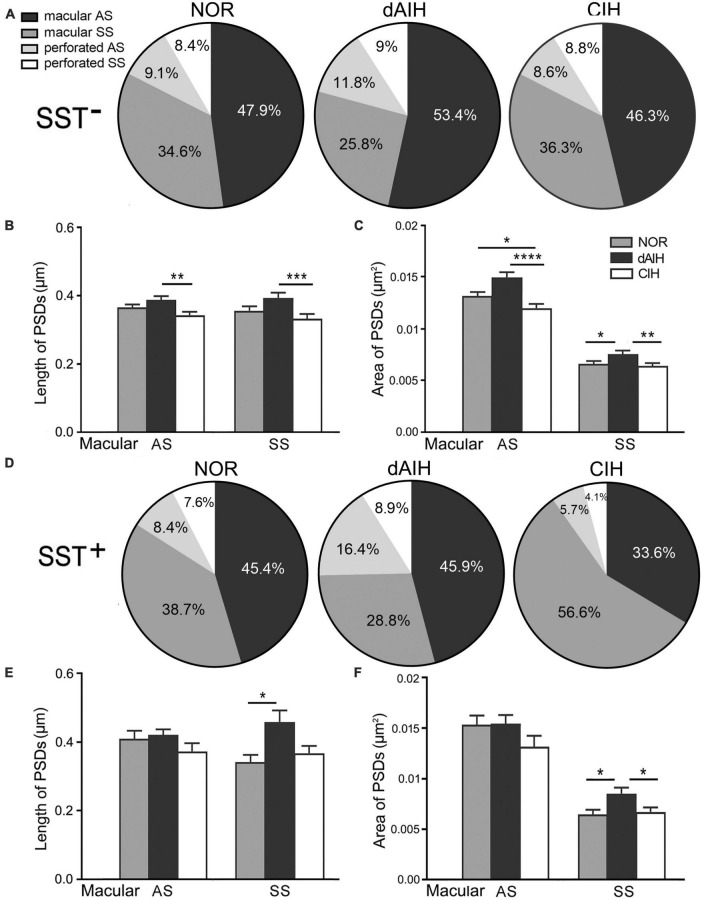
Percentages and the size of synapses formed by SST^–^ **(A–C)** or SST^+^ **(D–F)** terminals onto NK1R^+^ dendrites in the pre-BötC in the normoxic (NOR), daily acute intermittent hypoxia (dAIH) and chronic intermittent hypoxia (CIH) groups. Pie charts illustrated proportions of macular and perforated AS and SS **(A,D)**. The mean length of PSDs in SST^–^ AS and SS showed significant increases in the dAIH group, as compared to the CIH group (Kruskal–Wallis test, *p* = 0.0047 in AS, *p* = 0.0009 in SS), but had no significant differences while compared to the NOR group **(B)**. The mean area was also significantly increased in the dAIH group, as compared to the CIH group [**(C)** Kruskal–Wallis test, *p* < 0.0001 in AS, *p* = 0.0055 in SS]. Significant increases in the mean area were also identified in AS in the NOR group, as compared to the CIH group (Kruskal–Wallis test, *p* = 0.0159) and in SS in comparison with the dAIH group [**(C)** Kruskal–Wallis test, *p* = 0.0312]. Significant increase in the PSD size was identified only in SST^+^ SS in the dAIH group, as compared to the NOR [**(E,F)** Kruskal–Wallis test, *p* = 0.0249 in length; *p* = 0.0327 in area]. The mean area was also significantly increased in the dAIH group, as compared to the CIH group [**(F)** Kruskal–Wallis test, *p* = 0.021]. *p < 0.05, **p < 0.01, ***p < 0.001, ****p < 0.0001.

Synaptic characteristics were analyzed separately in terminals with or without SST immunoreactivity in the pre-BötC. Synapses with blurred clefts and PSDs due to the plane of sections were not included in analyses. A total of 1,543 synapses formed by SST^–^ terminals, including 514 in the NOR, 519 in the dAIH and 510 in the CIH groups were analyzed from over 60 grids derived from four brainstems (3–4 pre-BötC areas/brainstem, about 6–8 ultrathin sections/grid) in each group. Macular synapses accounted for a dominant proportion in the NOR (82.5%), dAIH (79.2%), and CIH (82.6%) groups, and the rest were perforated synapses (Figure 5A). The proportion of AS reached 65.2% in the dAIH, as compared to 57% in the NOR and 54.9% in the CIH groups. Conversely, macular SS were declined to 25.8% in the dAIH, as compared to 34.6% in the NOR and 36.3% in the CIH groups. Perforated synapses accounted for a higher proportion in the dAIH (20.8%) than in the NOR (17.5%) and CIH (17.4%) groups (Figure 5A). The mean length of macular synapses was significantly increased in the dAIH group, when compared to the CIH group (*p* < 0.01 in AS, *p* < 0.001 in SS), but had no significant difference when compared to the NOR group (Figure 5C). Significant increases in the mean area were found in the dAIH group, as compared to the NOR (*p* < 0.05 in SS) and the CIH (*p* < 0.0001 in AS, *p* < 0.01 in SS) groups, and also in the NOR, as compared to the CIH groups (*p* < 0.05 in AS, Figure 5C). Perforated synapses had much larger PSDs than macular ones, roughly by 70% (AS)–80% (SS) in length, and 60% (AS)–90% (SS) in area (Figure not showed).

### DLC ultrastructural features in the pre-BötC

**FIGURE 6:**
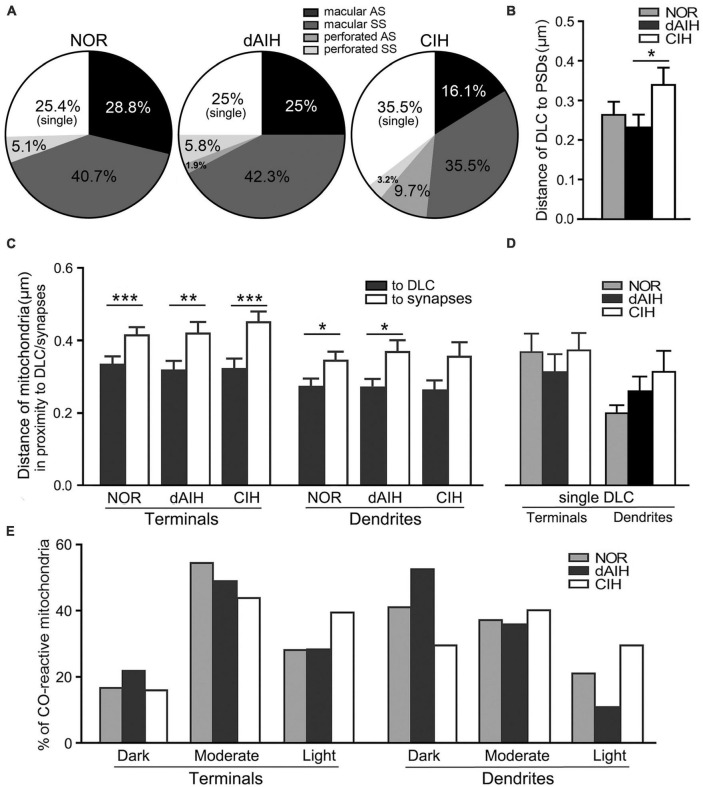
DLC relationships with synapses and mitochondria in the pre-BötC in the NOR, dAIH and CIH groups. Pie charts presented proportions of DLC in association with macular and perforated AS and SS **(A)**. DLC were in more proximity to synapses in the dAIH than the CIH group [**(B)** Kruskal–Wallis test, *p* = 0.035]. Mitochondria were found in more proximity to DLC than synapses in terminals [**(C)** Mann Whitney U test, *p* = 0.001 in NOR, *p* = 0.0059 in dAIH, *p* = 0.0004 in CIH] and in dendrites [**(C)** Mann Whitney U test, *p* = 0.023 in NOR, *p* = 0.019 in dAIH, *p* = 0.089 in CIH]. The distance of mitochondria to isolated DLC showed no significant differences of three groups **(D)**. Percentages of darkly, moderately and lightly CO-reactive mitochondria in proximity to DLC in terminals and dendrites were displayed in three groups **(E)**. *p < 0.05, **p < 0.01, ***p < 0.001.

DLC were identified for the first time in the pre-BötC (arrowheads in Figures 1E, F, 3, 4D, 8C, E). The DLC were originally described in Mauthner neurons in goldfish (Nakajima, 1974). They are localized adjacent to chemical synapses and gap junctions, and respond to LTP tetanic stimulations, thereby named mixed synapses together with chemical synapses and gap junctions that are assumed to represent chemical and electrical neurotransmission, respectively, occurring within the same terminal (Nakajima, 1974; Moshkov et al., 1998, Dzeban et al., 2004). DLC in the pre-BötC were featured by both prominent, dense membranes in apposition forming narrow clefts, which usually contained fibrous bridges, similar to synaptic clefts (solid thick arrowheads in Figures 3, 4D, 8C, E). Synaptic vesicles were frequently encountered in the DLC, and some were obviously accumulated, reminiscent of those in synapses (open arrowheads in Figures 3B–E, 4D, 8E). They were found alongside of AS (Figures 3F, 8C, E) or SS (Figures 1E, F, 3A–E, 4D), and some were resided between two segments of perforated synapses (solid arrowheads in Figures 1F, 3E). DLC seemed in more association with SS than AS. Dense membranes of DLC could be found in connection with PSDs, and fibrous bridges were continuous between them (Figures 1F, 3B–D). A total of 142 DLC (59 in NOR, 52 in dAIH, 31 in CIH) were examined in the pre-BötC. They were resided mainly alongside of synapses and with higher proportions in the NOR (74.6%) and the dAIH (75%) groups than in the CIH group (64.5%). The rest were in isolation with no synapse discernible in the same terminals (Figure 6A). They presented in more association with SS (45.8% in NOR, 48.1% in dAIH, 38.7% in CIH) than AS (28.8% in NOR, 26.9% in dAIH, 25.8% in CIH, Figure 6A). DLC were found more alongside of perforated synapses (12.9%), especially perforated AS (9.7%) in the CIH group than in the NOR (5.1%) and dAIH (7.7%) groups (Figure 6A). They were in more proximity to synapses in the dAIH than in the CIH group (*p* < 0.05), but had no significant difference, when compared to the NOR group (Figure 6B). There was also no significant difference in the distance of DLC to AS and SS, respectively, in three group (Figure not showed).

Of note, mitochondria appeared in greater proximity to DLC than synapses (open arrows in Figures 1E, 3, 4D with Figures 4 d2, 8C, E). Mitochondrial cristae were found in parallel toward DLC (open arrows in Figures 3A–E, 8C). Filamentous or flocculent elements could be seen between the mitochondria and the DLC (solid thin arrowheads in Figures 3A–C). A total of 240 mitochondria in terminals (95 in NOR, 77 in dAIH, 68 in CIH) and 194 ones in dendrites (75 in NOR, 72 in dAIH, 47 in CIH) were analyzed for the distance to DLC and synapses, respectively, and CO activity in the pre-BötC. Indeed, mitochondria were found in more proximity to DLC than synapses, especially in terminals (Figure 6C, *p* < 0.001 in NOR and CIH; *p* < 0.01 in dAIH). A similar trend was also identified in dendrites, although showing no significant difference in the CIH group (Figure 6C). The distance of mitochondrial positioning to DLC appeared to be more consistent with less variance in terminals and dendrites that had both DLC and synapses (Figure 6C) than those with isolated DLC only, although the later showed no significant difference (Figure 6D). High proportions of darkly CO-reactive mitochondria were identified in dendrites in the dAIH (52.8%) and NOR (41.3%) groups, where darkly to moderately reactive ones reached 88.9 and 78.6%, respectively. Moderately CO-reactive ones were prevalent in terminals (54.7% in NOR, 49.4% in dAIH, 44.1% in CIH). The highest proportions of lightly CO-reactive ones were evident in the CIH group, accounting for 39.7% in terminals and 29.8% in dendrites (Figure 6E).

### SST expression in the pre-BötC NK1R^+^ neurons

**FIGURE 7:**
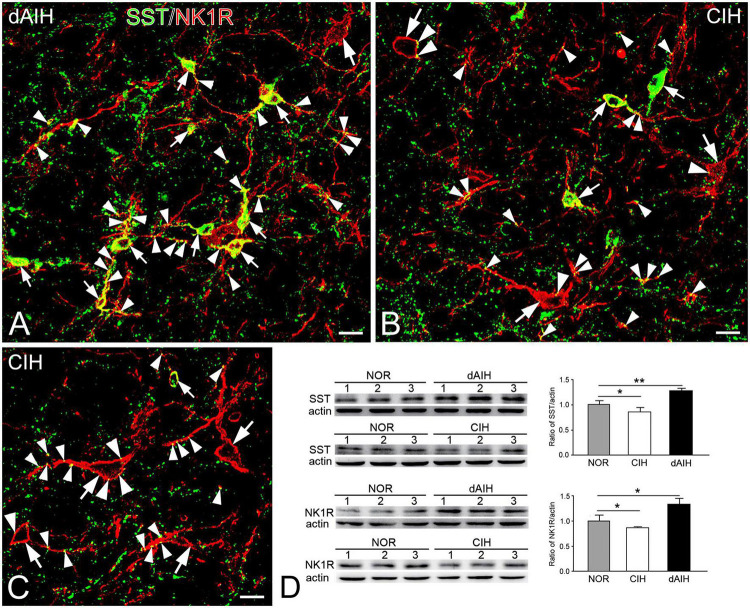
Expressions of SST and NK1R in the pre-BötC in the NOR, dAIH and CIH groups. SST immunoreactivity is visualized with Alexa 488 (green) and NK1R immunoreactivity with Texas Red (red). Thin arrows presented colocalization of two fluorophores in small fusiform neurons **(A–C)**. SST^+^ boutons were found in close association with NK1R^+^ somata (thick arrowheads) and processes (thin arrowheads). Thick arrows illustrated NK1R^+^ large neurons **(B,C)**. Western blots showing bands detected with antibodies against SST, NK1R and actin in tissues of the ventrolateral medulla oblongata, including the pre-BötC region **(D)**. Darker bands were found in lanes with protein extracts from the dAIH group, while lighter ones were from the CIH group. Scale bars: 20 μm, *p < 0.05, **p < 0.01.

SST was expressed in a group of small fusiform neurons that often overlapped with NK1R in the pre-BötC (Figures 7A, B). SST immunoreactivity appeared in somata and punctuate-like boutons, some of which were in close apposition to processes and somata that were NK1R^+^ (Figures 7A, B). Large NK1R^+^ neurons lacked SST immunoreactivity (Figures 7B, C). dAIH significantly increased expressions of SST and NK1R, whereas CIH led to a significant decline of both in the pre-BötC region (Figure 7D).

**FIGURE 8:**
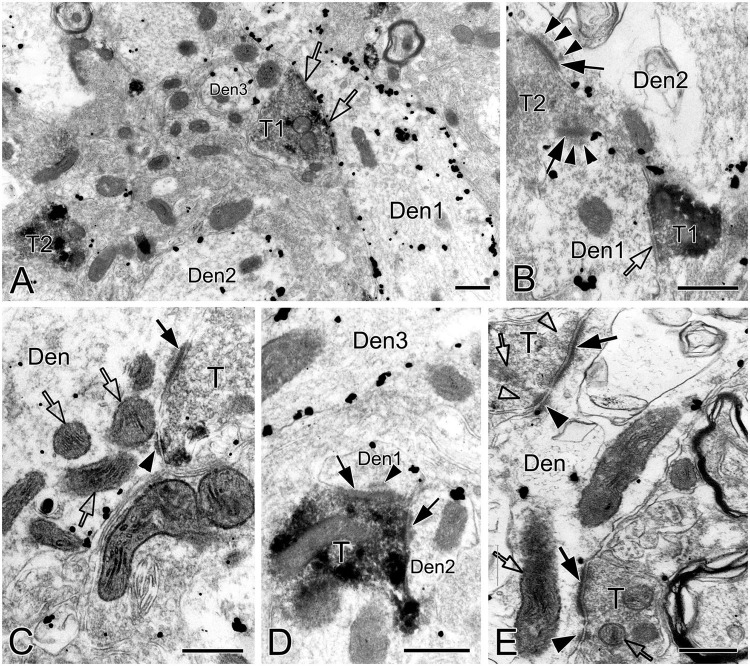
Electron micrographs showing AS, SS and DLC formed by SST^+^ terminals in the pre-BötC. SST^+^ terminals made SS [open arrows in panel **(A,B)**] and AS [solid arrows in panels **(C,D)**] onto NK1R^+^ dendrites. Arrowheads in panels **(B,D)** pointed to postsynaptic dense bodies. DLC were found alongside of AS [arrowheads in panels **(C,E)**]. Mitochondria could be seen in proximity to DLC [open arrows in panels **(C,E)**]. Synaptic vesicles appeared in accumulation in distinct pools opposed to PSDs and DLC [open arrowheads in panel **(E)**].

**FIGURE 9:**
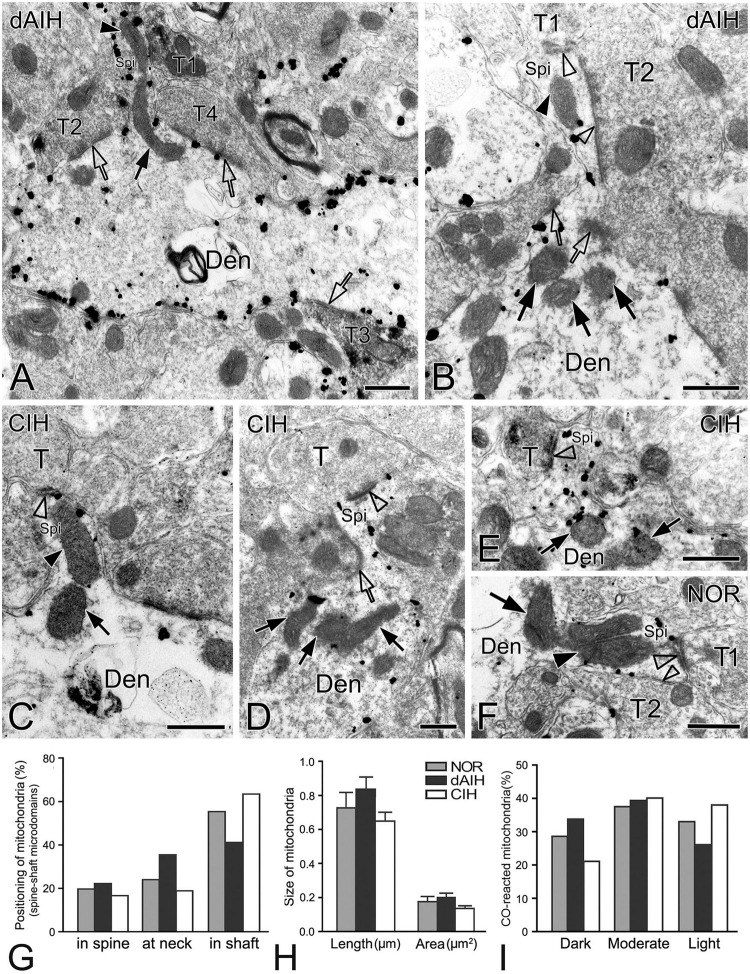
Electron micrographs and histograms showing spines and associated mitochondria in the pre-BötC in three groups. Spine-shaft microdomains presented synapses in spines [synapse with T1 in panel **(A)**, open arrowheads in panel **(B)**] and in parent shafts adjacent to the base of the spine neck [open arrows in panels **(A,B)**], which were coupled with mitochondria positioning in spines [solid arrowhead in panels **(A,B)**], at the spine neck [solid arrow in panel **(A)**] and in parent shafts near the base of the spine neck [solid arrows in panel **(B)**]. Multiple-synapse spines with both AS and SS were detectable [open arrowheads in panels **(B,F)**]. A presumed SS at the spine neck [open arrow in panel **(D)]** extended into a spine that also received an AS on the head [open arrowhead in panel **(D)**]. Mitochondria seemed successively in spines [solid arrowheads in panels **(C,F)**] with a follower at the spine neck [arrows in panels **(C,F)]**. SST^+^ terminals made synapses onto spines [synapse with T1 in panel **(A)**, open arrowhead in panel **(E)**], in the spine-shaft microdomain [open arrow with T2 in panel **(A)**], and on the shaft [open arrow with T3 in panel **(A)**]. T: terminal, Den: dendrite, Spi: spine. Scale bars: 0.5 μm. Histograms showed mitochondrial positioning **(G)**, size **(H)** and co reaction **(I)** in spine-shaft microdomains.

SST immunoreactive product displayed an uneven flocculent or spot-like appearance when colocalized with NK1R in somata (arrowheads in Figure 1A, B) and primary dendrites (arrowheads with Den in Figure 1B) under the electron microscope. In terminals, SST typically resided within large dense-core vesicles (Figure 1D with T2, Figure 8A with T1); however, it could be diffuse throughout the terminals, depending on the extent to which sections were exposed to the DAB reaction, as exemplified in Figure 2C with T2, D. SST^+^ terminals made AS or SS on NK1R^+^ dendritic shafts (Figure 2C with T2, Figures 4A, 8A, B with T1, Figures 4C, D, 9A with T2, T3), spines (Figure 9A with T1, E), and somata (Figure 1D). An SST^+^ terminal could be found to form SS (arrowheads in Figure 2C) or AS (arrows in Figure 8D) with two NK1R^+^ dendrites.

A total of 387 synapses (119 in NOR, 146 in dAIH, 122 in CIH) established by SST^+^ terminals were analyzed in the pre-BötC. AS accounted for the highest proportion (62.3%) in the dAIH and the lowest (39.3%) in the CIH, as compared to the NOR group (53.8%). Perforated synapses were in the highest proportion of 25.3% (16.4% AS, 8.9% SS) in the dAIH group, as compared to 16% in the NOR and 9.8% in the CIH groups (Figure 5D). In contrast, macular SS were prevalent in the CIH (56.6%, Figure 5D). Significant increases in the mean length and area of PSDs were found only in macular SS in the dAIH group, as compared to the NOR group (Figures 5E, F, *p* < 0.05), and also in the mean area in the dAIH group, as compared to the CIH group (Figure 5F, *p* < 0.05).

### Ultrastructural characteristics of the spine-shaft microdomain in the pre-BötC

Identification of spines requires that the spine must be visually traced to the parent dendrite, which is uncommon and also a challenge under the electron microscope that the section passes through both the head and the neck connecting it to the parent dendrite. However, three categories of dendritic spines described in previous reports (Peters and Kaiserman-Abramof, 1970; Harris and Kater, 1994) were defined in the pre-BötC, including mushroom spines with a narrow neck and a large head (Figures 9D, E), thin spines with a slender neck and a smaller head (Figure 9A), and stubby spines with the width of the neck similar to the length (Figure 9C). We found multiple-synapse spines composed of one AS (thick open arrowheads in Figures 9B, F) and one SS (thin open arrowheads in Figures 9B, F) in the pre-BötC. SST^+^ peptidergic modulatory synapses were also identified in spines (Figure 9A with T1, E). Figure 9D illustrates a presumed SS formed by a peptidergic terminal containing large dense-core vesicles, positioning partly in the spine and partly in the parent shaft (open arrow), and a AS on the head (open arrowhead) for seeming excitatory-inhibitory modification in a single spine. Synapses in parent shafts adjacent to the base of the neck could be seen in oblique distribution toward spines (open arrows in Figures 9A, B), some of which even extended into spines through the neck (open arrow in Figure 9D), establishing a structural base, characterized as the spine-shaft microdomain for the spine and shaft communication. Figure 9A presents three SST^+^ terminals in distinct synaptic formations: one AS on the spine (T1), one shaft SS in the spine-shaft microdomain (T2), and another SS on the shaft (T3), indicating SST-mediated diverse synaptic modifications.

Mitochondria presented tight correlation with synapses in spine-shaft microdomains in the pre-BötC. We found for the first time that mitochondria were within spines in the pre-BötC (solid arrowheads in Figures 9A–C, F). Some were distributed partly in spines and partly in parent shafts (solid arrows in Figures 9A, C, F), and some were in parent shafts near the base of the spine neck (solid arrows in Figures 9B, D, E). Figure 9F displays three mitochondria, two in parallel in a multiple-synapse spine (solid arrowhead), and another at the neck of the spine (arrow). Of 145 mitochondria (45 in NOR, 53 in dAIH, 47 in CIH) examined in 90 spine-shaft microdomains (28 in NOR, 31 in dAIH, 31 in CIH), a majority of mitochondria (58.5%) were found positioning completely or partly in spines in the dAIH group, whereas they were predominantly in parent shafts in the NOR (55.5%) and CIH (63.8%) groups (Figure 9G). There were no significant differences in the mean length and area of mitochondria of three groups (Figure 9H). Most mitochondria were darkly to moderately CO-reactive in the NOR (66.7%) and dAIH (73.6%) groups, whereas moderate to light ones (81.4%) were predominant in the CIH group (78.7%, Figure 9I), indicating increased mitochondrial activity induced by dAIH.

### Ultrastructural characteristics of mitochondrial fusion and fission in the pre-BötC

**FIGURE 10:**
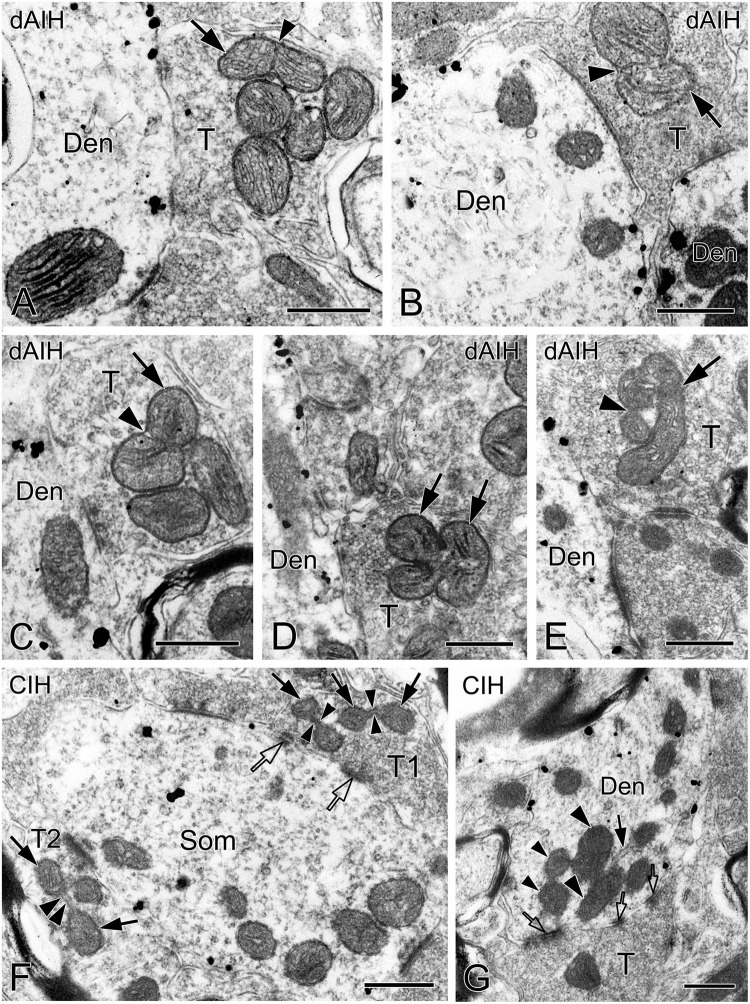
Electron micrographs showing mitochondrial fusion and fission in the pre-BötC. Fusion was identified between two larger mitochondria [arrows in panels **(A–D)**]. The fused outer membrane was clearly visible with a slight curvature toward the inner membrane [arrowheads in panels **(A–C)**]. Fission was found between two smaller mitochondria [arrows in panel **(F)**] interconnected by a mitochondrial tubule [arrowheads in panel **(F)**]. A cluster of mitochondria overlapped in close proximity to a perforated synapse [open arrows in panel **(G)**], of which the larger two (thick arrowheads) seemed in fusion, and the smaller two (thin arrowheads) in fission. The endoplasmic reticulum was commonly visible among mitochondria [solid arrow in panel **(G)]**. Filamentous or flocculent elements could be found between mitochondria [arrowhead in panel **(E)**]. Den: dendrite, T: terminal. Scale bars: 0.5 μm.

We defined distinct ultrastructural morphologies of mitochondrial fusion and fission, which were much less known in *in vivo* animal studies, and lacking in those on the pre-BötC. Morphological features of mitochondrial fusion were characterized as three steps, based on our observations: first, the outer membrane of two apposed mitochondria interconnected and fused with a slight curvature toward the inner membrane, forming a narrow intermembrane space (arrowheads in Figures 10A–C). Second, the two inner membranes were in close apposition to enlarge the membrane contact area, where dot-like contacts could be visualized (Figures 10A, B). The inner membranes fused partly (Figures 10B, C) to completely (Figure 10D), leading to an elongated and enlarged mitochondrion (Figures 10A–D). Third, the cristae appeared in rearrangement, seemingly perpendicular toward (Figures 10A, B) or across the fused inner membranes (Figures 10C, D). Ultrastructural characteristics of mitochondrial fission were identified between two smaller mitochondria (solid arrows in Figure 10F) that were linked by a mitochondrial tubule (arrowheads in Figure 10F), as described in an *in vitro* study (Basu et al., 2017). However, morphologies of mitochondrial fusion or fission were not always clearly visible because of the obscured membrane due to ultrathin sectioning (thin arrowhead in Figure 2A, arrowhead in 10E). Figure 10G displays a cluster of dendritic mitochondria in close proximity to a perforated synapse (open arrows), of which the larger two (thick arrowheads) seem to be in fusion with dark cristae interconnected between them, whereas the smaller two (thin arrowheads) could be in fission.

## Discussion

The pre-BötC is a heterogeneous network, containing nearly half excitatory glutamatergic neurons expressing the developing brain homeobox 1 (Dbx1^+^) and half inhibitory GABAergic and glycinergic neurons (Kuwana et al., 2006; Wallén-Mackenzie et al., 2006; Winter et al., 2009). SST and NK1R partially co-localize with glutamatergic Dbx1^+^ neurons (Cui et al., 2016; de Sousa Abreu et al., 2022). Inspiratory rhythm generation relies on a synchronous convergence of glutamatergic excitatory drive (Smith et al., 1991; Gray et al., 2001; Feldman et al., 2013), whilst inhibitory neurons play a critical role in shaping breathing movements by mediating apneas, stabilizing the rhythm, and broadening the dynamic range of inspiration, endowing the rhythm with flexibility to adapt to environmental, metabolic, and behavioral needs (Cui et al., 2016; Baertsch et al., 2018; Ashhad and Feldman, 2020). In the present study, we found that first, dAIH induced significant increases in the PSD size of macular AS and the proportion of perforated synapses in the pre-BötC. dAIH shifted excitation-inhibition balance toward a higher excitation with dominant AS in shafts, whereas CIH led to a higher inhibition with increased SS, especially SS formed by SST^+^ terminals. Second, DLC were featured for the first time in the pre-BötC. They were localized mainly alongside of synapses, especially SS, and in more proximity to them in the dAIH than CIH group. Mitochondria appeared in more proximity to DLC than synapses of three groups. Third, multiple synapses with dual AS and SS innervation, including peptidergic SST^+^ synapses, were identified on the same spines. Spine-shaft microdomains established by synapses in spines and parent shafts coupled with mitochondrial positioning was characterized for the first time in the pre-BötC, which may form the basis for synchronizing spine-shaft communications. Finally, we depicted ultrastructural features of mitochondrial fusion and fission in the pre-BötC for the first time. We provided ultrastructural evidence that synapses and DLC coupled with mitochondrial activity are closely associated in synapse-specific compartments in the pre-BötC, most likely participating in respiratory plasticity.

### Ultrastructural alterations of SST^–^ synapses in dendritic shafts

Synaptic ultrastructural alterations correlate with functional activity of synapses. In cortical and hippocampal synapses, the shape and surface area of PSDs directly correlate with neurotransmitter release probability, synaptic strength, efficiency, and plasticity (Holderith et al., 2012; Eltokhi et al., 2020; Holler et al., 2021). The PSD surface area is proportional to the number of postsynaptic receptors, the larger the surface of the PSD, the higher the absolute number of AMPARs and NMDARs (Kharazia and Weinberg, 1999; Tarusawa et al., 2009). Perforated synapses were first described in rat cerebral cortex by Peters and Kaiserman-Abramof (1969), characterized by their large size and discontinuity of the PSDs when sectioned passing at right angles to synapses. Their presence increases the extent of the edge of PSDs and hence the size of the total synaptic active zone (Peters and Kaiserman-Abramof, 1969; Jones and Harris, 1995). The considerably large PSDs of perforated synapses have more receptors to be facilitated for synaptic transmission in correlation with synaptic plasticity and long-lasting memory (Toni et al., 2001; Ganeshina et al., 2004; Stewart et al., 2005; Spruston, 2008). During chemically induced LTP, macular PSDs are able to transform into perforated PSDs, to facilitate PSD expansion by adding new molecules from peri-synaptic areas (Stewart et al., 2005). Perforated synapses can be identified in single section, stereological analyses, or 3D reconstruction that were then classified as fenestrated, horseshoe-shaped, and segmented shapes (Geinisman et al., 1987; Harris et al., 1992). However, the present study was performed on 50 μm thick sections with preembedding immunoreactivities that labeled surface neurons within a few microns in-depth, thereby limiting 3D analyses. Indeed, SST and NK1R double immunoreactivity precisely delineated AS and SS, of whose perforated synapses were characterized for the first time in the pre-BötC. Perforated synapses presented remarkably larger PSDs than macular synapses. dAIH increased the proportion of perforated synapses, especially in AS, implying increased synaptic transmission probability and synaptic strength. The finding of synaptic vesicles accumulated in distinct pools in apposition to each discrete PSD segments suggests that they may function as independent units of each segment, equivalent to increasing the number of release sites, to enhance synaptic transmission efficacy (Toni et al., 2001; Ganeshina et al., 2004). Consistently, the structure and composition of PSDs are dynamic, which can be rapidly modified in alignment with presynaptic release sites, so called as nanocolumns (Tang et al., 2016). Cryo-electron tomography reveals trans-synaptic assemblies that link presynaptic vesicles with putative postsynaptic receptors, bridging the cleft for trans-synaptic coordination of synaptic transmission and plasticity (Martinez-Sanchez et al., 2021). Fibrous bridges examined in synaptic clefts in the pre-BötC may play a role, at least, as a structural basis for the trans-synaptic coordination and plasticity. Moreover, fibrous bridges were also identified in DLC resided alongside of synapses and some were continuously connected with synaptic clefts, as represented in Figures 3C, D, which may be morphological evidence of DLC in synchrony with synaptic activity that respond to respiratory plasticity.

The balance of synaptic excitation-inhibition is a crucial determinant of network output, including pre-BötC neurons (Baertsch et al., 2018; Ashhad and Feldman, 2020). While dAIH induced a significant increase in the PSD size of macular AS, it was increased as well in the macular SS, implying a synchronized excitation-inhibition balance with appropriate inhibition in modulation of the network augmented plasticity. Alterations in AS and SS compositions with dominant AS in the dAIH group and high SS proportion in the CIH group suggest excitation-inhibition balance shifting toward a higher excitation associated with LTF expression and a higher inhibition associated with OSA. A moderate dAIH preconditioning may stimulate glutamatergic microcircuits in the pre-BötC that increase Ca^2+^ entry through NMDA receptors, and in turn, recruit AMPA receptor insertion into synapses, thereby enlarging the PSD size and the number of AS for augmented respiratory plasticity. Conversely, severe CIH challenges induce excessive Ca^2+^ influx and increased ROS generation (Semenza and Prabhakar, 2015), leading to calcium overload and oxidative stress that may render the network unstable and less plastic with a robust inhibition and apnea in association with detrimental consequence of OSA.

### DLC relationships with synapses

DLC are assumed as a composition of mixed synapses with chemical synapses and gap junctions, responding to tetanization-induced LTP in Mauthner neurons in goldfish (Moshkov et al., 1998, Dzeban et al., 2004). DLC consist predominantly of filamentous actin, which possesses electroconductivity, and is well-known to be involved in mechanisms of LTP and synaptic plasticity (Pinho et al., 2020). Fibrous bridges in the clefts of DLC are associated with polymerizd actin, which may be the structural substrate of electrotonic transmission (Dzeban et al., 2004), functioning directly via shunting chemical synapses, or indirectly through interaction with gap junctions in response to synaptic plasticity (Moshkov et al., 1998). Indeed, DLC examined in the pre-BötC manifested a close association with synapses, especially SS, even that dense membranes of DLC and PSDs, as well as fibrous bridges within clefts were connected continuously, implying possible chemical synapses and DLC electroconductive interactions. The interactions could be more intimate under dAIH challenge, given that DLC appeared more alongside of synapses and were in more proximity to them in the dAIH group than in the CIH group. If considering DLC in involvement in modulating the balance of synaptic excitation-inhibition in the pre-BötC, as described above, they could be more in coordination with inhibitory synapses under dAIH challenge, to maintain an appropriate augmentation, as near half of DLC stayed adjacent to SS that were almost double the amount of DLC associated with AS in the dAIH group. On the contrary, DLC may turn to a close association with perforated AS in synergism with synaptic excitation in the CIH group, while the balance of excitation-inhibition tends to shift toward inhibition exerted by CIH challenge. Our ultrastructural findings implicate the network communications between DLC and synapses, macular and perforated synapses, and excitation and inhibition balance in the pre-BötC in their contribution to respiratory plasticity, which however, remain to be explored.

Mitochondria positioning in more proximity to DLC than synapses reflect robust activity of DLC that needs high energy supply as synaptic property rather than a simple contact. Higher proportion of darkly CO-reactive mitochondria in dendrites than terminals suggests a higher energy demand at postsynaptic compartments than presynaptic compartments, where moderately CO-reactive ones were predominant. The energy requirement could be stable and continuous for DLC in synchrony with synaptic activity, thereby corresponding to a more consistent mitochondrial positioning to DLC in terminals and dendrites that had both DLC and synapses than those with isolated DLC only. If they should be named mixed synapses as described in the Mauthner neurons, they are composed of chemical synapses and DLC only, as we have not found gap junctions between neurons, except ones between glial cells in the pre-BötC (Kang et al., 2017). Their colocalizations may indicate chemical and electrotonic neurotransmission and interaction occurring within the same terminal, which, however, remains to be investigated. DLC in cone pedicles in the retina of macaque monkeys do not express desmosome proteins, such as desmocollin, desmoplakin, plakoglobin, pan-cadherin and catenin (Paffenholz et al., 1999), but are associated with glutamate receptors (Haverkamp et al., 2000). Whether DLC identified in the pre-BötC express distinct receptors and neurotransmitters remains to be explored.

### Ultrastructural alterations of SST^+^ synapses in dendritic shafts

The pre-BötC neurons receive both SST^+^ AS and SS innervation (Wei et al., 2012; Cui et al., 2016). SST^+^ AS inputs are most likely from glutamatergic pre-BötC neurons (Cui et al., 2016; de Sousa Abreu et al., 2022). SST^+^ SS inputs may arise mainly from sources outside the pre-BötC, such as the nucleus of the solitary tract, Kölliker-Fuse nucleus, or the parabrachial nuclei that contain SST^+^ inhibitory neurons projecting onto the pre-BötC (Epelbaum et al., 1994; Bou Farah et al., 2016). The colocalization of SST with glutamate and their probable corelease imply that SST may prevent the network from overexcitation to maintain a proper rhythm stability in a phase- and state-dependent manner (Ramírez-Jarquín et al., 2012). In the present study, the proportion of SST^+^ AS was remarkably increased in the pre-BötC in the dAIH group. It is possible that terminals previously with less or undetectable SST immunoreactivity acquired recognizable amount of SST to maintain the augmented glutamatergic stability. Conversely, SST^+^ SS were predominant in the CIH group. An increase in the PSD size in macular SS, not AS, induced by dAIH implicates an inhibitory compensatory mechanism in maintaining excitation within certain limits and stability (Cui et al., 2016; Baertsch et al., 2018). SST, as an inhibitory neuromodulator, directly depresses pre-BötC-generated rhythms (Ramírez-Jarquín et al., 2012). SST modulation is required for proper gasping, as the blockade of SST receptor 2 compromises auto-resuscitation (Ramírez-Jarquín et al., 2012). SST is also found decline in the pre-BötC in victims with sudden infant death syndrome (Lavezzi and Matturri, 2008). Reduction of SST expression exerted by CIH in the pre-BötC may be relevant to gasping dysfunction, which, however, remains to be determined.

### Spine-shaft microdomains in the pre-BötC

Both excitatory and inhibitory inputs are involved in fine-tuning synaptic integration and plasticity in dendritic shafts and spines (Chen et al., 2012; Chiu et al., 2018). It is reported that excitatory synapses on dually innervated spines are more stable than those on singly innervated spines. Inhibitory synapses are more dynamic than excitatory synapses on the same dually innervated spines, thereby excitatory synapses are almost exclusively one-time events of removal and addition, whereas inhibitory synapses are frequently transient or recurrent in the same location, manifesting substantial and dynamic rearrangements of GABAergic synapses in mediating synaptic plasticity (Chen et al., 2012; Villa et al., 2016). GABAergic PSDs, similar to glutamatergic synapses, enable rapid diffusion in the plasma membrane to disperse synaptic GABA receptors, or conversely, transiently interact with scaffold proteins for synaptic accumulation (Bannai et al., 2015; Bertholet et al., 2016). GABAergic synapses in the cortex can directly impinge onto glutamatergic spines, exerting highly compartmentalized control of excitation (Chen et al., 2012; Chiu et al., 2013; Villa et al., 2016). Multiple-synapse spines with dual AS and SS innervation in the pre-BötC were found in the pre-BötC. The positioning of SS partly in the shaft and partly in the spine, as exemplified in Figure 9D may represent a highly dynamic state that SS at the neck are processing insertion or dispersal of inhibitory receptors in the postsynaptic membrane in synchrony with excitatory synaptic activity within a single spine. The spine-shaft microdomain characterized by accumulated synapses in spines, at the neck, and in parent shafts, coupled with mitochondrial positioning provides a structural evidence for the spine-shaft communication. Our finding is consistent with understanding of functional short-range crosstalk between the spine and the parent shaft that excitatory plasticity at an individual spine can affect neighboring GABAergic synapses in the parent shaft, and further likely to influence dendritic information processing and integration (Ravasenga et al., 2022).

### Mitochondrial dynamics in the spine-shaft microdomains and their fusion and fission in the pre-BötC

Mitochondria are highly dynamic in structure and function in correspondence with respiratory plasticity elicited by dAIH and CIH in dendrites in the pre-BötC (Kang et al., 2019, 2020). In the present study, we found that mitochondria in spine-shaft microdomains were positioned diversely within spines, partly in spines and partly in shafts, or in shafts adjacent to the base of the spine neck. dAIH elicited more mitochondria extending into the spine, implying high energy expenditure of synapses for augmented LTF expression. Mitochondrial recruitment to synapses is associated with local Ca^2+^ elevation during sustained synaptic activities (Seager et al., 2020). dAIH-induced mitochondrial positioning in spines could be attributed to local elevated Ca^2+^ influx that facilitates mitochondrion-spine communication. Conversely, the majority of mitochondria with light CO reaction stayed in parent shafts in the CIH group, which may be interpreted as CIH-elicited Ca^2+^ overload and ROS increase that negatively impact CO activity and mitochondrial entrance into spines.

Mitochondria are in a perpetual state of fusion and fission to maintain their structure, quality, and function (Seager et al., 2020). An imbalance of these two opposite processes, particularly an excess of fission can be detrimental to mitochondrial function (Fischer et al., 2016). We found that mitochondrial fusion appeared between larger mitochondria with dark CO reactivity, being consistent with a robust mitochondrial activity, whereas fission was observed between two smaller mitochondria. The structural evidence will guide a functional understanding of mitochondrial fusion and fission associated with respiratory plasticity.

In summary, the present study demonstrates synaptic ultrastructural alterations in the pre-BötC induced by dAIH and CIH. Alterations in AS and SS composition, the PSD size, and the number of perforated synapses implicate a shift of excitation-inhibition balance toward a higher excitation under dAIH challenge, and conversely, a higher inhibition induced by CIH. The excitation-inhibition network is precisely displayed in spine-shaft microdomains, such that multiple-synapse spines have dual AS and SS innervation, and SS at the spine neck extend into spines for crosstalk with AS in spines, establishing structural evidence for understanding spine-shaft communication. The close association of DLC with synapses may imply chemical and electrotonic neurotransmission and interactions occurring within the same terminal, which are in synchrony with mitochondrial dynamics in structure, positioning and CO activity. Our ultrastructural evidence highlights the excitation-inhibition network in shafts and spines, and DLC in association with synapses that coincide with mitochondrial dynamic in their contribution to respiratory plasticity in the pre-BötC.

## Data availability statement

The original contributions presented in this study are included in the article/supplementary material, further inquiries can be directed to the corresponding author.

## Ethics statement

The animal study was reviewed and approved by the Northwest China Committee of Experimental Animal Care.

## Author contributions

Y-YL designed and performed the experiments, and wrote the manuscript. JK, NL, and SY performed the research. BG and YZ contributed hypoxic animal preparations. SW and XH analyzed the data. MW-R reviewed the manuscript. All authors contributed to the work and approved the submitted version.
